# Synergistic Interaction between the Entomopathogenic Fungus *Akanthomyces attenuatus* (Zare & Gams) and the Botanical Insecticide Matrine against *Megalurothrips usitatus* (Bagrall)

**DOI:** 10.3390/jof7070536

**Published:** 2021-07-03

**Authors:** Jianhui Wu, Bo Yang, Xiaochen Zhang, Andrew G. S. Cuthbertson, Shaukat Ali

**Affiliations:** 1 Key Laboratory of Bio-Pesticide Innovation and Application, College of Plant Protection, South China Agricultural University, Guangzhou 510642, China; jhw@scau.edu.cn; 2 Engineering Research Center of Biological Control, Ministry of Education and Guangdong Province, South China Agricultural University, Guangzhou 510642, China; yb@stu.scau.edu.cn (B.Y.); zhangxiaoying@stu.scau.edu.cn (X.Z.); 3 Independent Science Advisor, York YO10 5AQ, UK; andrew_cuthbertson@live.co.uk

**Keywords:** entomopathogenic fungi, biopesticides, matrine, *Megalurothrips usitatus*, synergistic effect

## Abstract

The excessive use of synthetic chemicals for *Megalurothrips usitatus* (Bagrall) management has resulted in the development of insecticide resistance as well as adverse effects to the natural ecosystem. This has driven the need to develop alternative pest control strategies. This study reports a synergistic interaction between the entomopathogenic fungus *Akanthomyces attenuatus* (Zare & Gams) and the botanical insecticide matrine against *M. usitatus*. The results revealed that the germination rate and colony growth of *A. attenuatus* were inhibited by higher matrine concentrations. Percentage mortalities of *M. usitatus* following application of *A. attenuatus* and matrine showed a dose mortality effect. After five days of treatment, all concentrations of matrine combined with different concentrations of *A. attenuatus*, except one combination (matrine 0.25 mg/mL + 1 × 10^7^ conidia/mL), showed synergistic effect. The activities of acetylcholinesterase and antioxidant enzymes (superoxide dismutase, catalase and peroxidase) in *M. usitatus,* in response to individual or combined application of *A. attenuatus* and matrine at the end of the experimental period, were significantly lower than controls. The findings confirm the synergistic action of *A. attenuatus* and matrine against *M. usitatus* along with the biochemical phenomenon possibly regulating the synergistic effect.

## 1. Introduction

*Megalurothrips usitatus* (Bagnall) (Thysanoptera: Thripidae) is a primary pest of cowpea in China. This insect pest damages the leaves, flowers and pods leading to reduced yield as well as quality of cowpea [1,2,3]. At present, the control of *M. usitatus* mainly relies on chemical measures. Due to the small size of thrips and their cryptic nature, short development period, strong fecundity, parthenogenetic reproduction, pupation and the strong migration ability of adults, the use of chemicals is also inefficient in the control of thrips [3]. The excessive use of chemicals can also cause insecticide resistance and harmful risks from pesticide residues [2,4]. In the pursuit of environmentally friendly control strategies, entomopathogenic fungi have become an important means of biological control of invertebrate pests because of their wide host spectrum, strong pathogenicity, and environmental friendliness [3,5,6,7].

*Akanthomyces attenuatus* (Zare & Gams) (Hypocreales; Cordycipitaceae) (previously *Lecanicillium attenuatus*), is known to have pathogenicity against different insect pests [3,7]. The efficiency of the fungus as a control agent is dependent upon numerous biological events which are initiated by the adhesion of fungal spores to the insect cuticle, spore germination, and hyphal growth. In order, then, to breach the insect cuticle, fungal hyphae exert mechanical pressure and produce different enzymes and secondary compounds [5]. *Akanthomyces attenuatus* produces a secondary metabolite called bassianolide (a cyclooligomer depsipeptide), which can affect acetylcholine (Ach) receptors of insect muscles, reducing the production of acetylcholinesterase (AChE). Du et al. [7] showed 76.25 and 57.5% *M. usitatus* mortality and LC_50_ values of 1.9 × 10^6^ and 1.5 × 10^7^ conidia/mL following the application of two newly identified isolates of *A. attenuatus*: SCAUDCL-38 and SCAUDCL-56, respectively.

Matrine is a natural plant pesticide obtained from *Sophora flavescens* and *Sophora alopecuroides* roots [8]. Matrine has shown medicinal activities ranging from being antibacterial and anti-inflammatory, to liver protection and diuretic effects. Matrine has also been used to control different insect pests of various crops [9]. Matrine is known to target insect acetylcholine receptors, which in turn affects acetylcholinesterase production [10]. Matrine has shown antifeedant activities against Formosan subterranean termites (*Coptotermes formosanus* Shiraki) and two spotted spider mites (*Tetranychus urticae* Koch) [8,11]. Wu et al. [12] showed that different matrine treatments caused a dose dependent increase in *Spodoptera litura* mortality at different time intervals. Hwang et al. [13] described the efficacy of a chemical formulation (KNI3126) based on a mixture of matrine and neem oil against different sucking insect pests and phytophagous mites, thus confirming the toxic as well as biological action of matrine against phytophagous arthropods with different feeding habits. Matrine has the characteristics of specificity and naturalness [14]. It can only act on specific organisms and decomposes rapidly in nature as carbon dioxide and water. Due to these characteristics, matrine is considered as a green/environmentally friendly insecticide [15]. Matrine has been commercialized under different trade names; however, its insecticidal activity is lower than the popular insecticides introduced by international pesticide companies during the last few years [10].

Insects have a well-developed defense mechanism against insecticides and natural pathogens consisting of multiple enzyme systems [16]. Detoxifying enzymes, such as acetylcholinesterase and antioxidant enzymes (SOD, CAT, POD), are mainly responsible for insect defense to outer challenges caused by chemicals or pathogens [17,18]. Previous studies have also reported significant changes in enzyme activities following insecticide poisoning and fungal infection [19,20]. Only a little work has been performed to observe the complex changes in enzyme profiles following joint application of insecticide and pathogens against different insect pests [16,18]. However, a detailed investigation on the effects of the synergistic action between *A. attenuatus* and matrine on *M. usitatus* enzyme systems has not been reported.

This study aims to determine a possible synergism between matrine and *A. attenuatus* for *M. usitatus* management since both of these agents have the same site of action within their host [21,22]. In our study, the efficacy of different concentrations of matrine combined with various conidial concentrations of *A. attenuatus* were tested against *M. usitatus* (Bagnall). The effects of matrine or *A. attenuatus* application alone or in combination on enzyme activities of *M. usitatus* were also studied. The overall objective of this study was to provide a scientific basis for the synergistic effect of pathogenic fungi (*A. attenuatus*) and botanical pesticides (Matrine) for *M. usitatus* management.

## 2. Materials and Methods

### 2.1. Rearing of Akanthomyces attenuatus

Bean flower thrips adults collected from experimental fields at South China Agricultural University, Guangzhou were reared on bean pods under laboratory conditions (26 ± 5 °C; 70 ± 5% R.H and 12 h:8 h (light:dark) photoperiod) following the methods of Espinosa et al. [23] and Du et al. [7].

### 2.2. Fungal Culture and Preparations of Conidial Suspension

The *Akanthomyces attenuatus* isolate SCAUDCL-53 used for this study was originally isolated from soil samples and identified through molecular characterization of its ITS region by Yang et al. [3]. The isolate has been deposited in the Guangdong Microbial Culture Center under repository number 60586. The fungal cultures and basal conidial concentration (1 × 10^8^ conidia/mL) were prepared as per the method described by Ali et al. [22]. Briefly, the *A. attenuatus* isolate SCAUDCL-53 was cultured on potato dextrose agar (PDA) plates for 10 days under laboratory conditions. Conidia were harvested by scraping the surface of the mycelia with sterile cell scrapers into sterile de-ionized water containing 0.05% Tween-80. The conidia were counted under a compound microscope (Ningbo Shunning Instruments Co. Ltd., Ningbo, China) at ×40 magnification using a hemocytometer (Qian Yihua Glass Instruments Co. Ltd. Guangzhou, China) to calibrate a concentration of 1 × 10^8^ conidia mL^−1^. Lower concentrations of conidial suspension (1 × 10^7^, 1 × 10^6^, 1 × 10^5^, and 1 × 10^6^ conidia/mL), to be used in subsequent trials, were prepared via the serial dilution method.

Spore viability was determined before preparation of suspension by spreading 0.2 mL of a 1 × 10^4^ conidia mL^−1^ suspension on PDA, and the number of germinated propagules were estimated under a compound microscope (Ningbo Shunning Instruments Co. Ltd., Guangzhou, China) at 40× magnification after 24 h of incubation at room temperature. Spores were considered viable when the germ tube lengths corresponded to the width. The viability of conidia was assessed immediately before each experiment was started, and percentage germination was estimated to >95% for all experiments [24].

### 2.3. Matrine

Matrine (98% pure) was obtained from Guangdong New Scene Bioengineering Company Ltd., Yangjiang, P.R. China. A stock solution of matrine (5 mg/mL) was prepared using sterile ddH_2_O as solvent. Lower concentrations of matrine (1.0, 0.5, 0.25, 0.125, 0.05 mg/mL), to be used in subsequent trials, were prepared via the serial dilution method.

### 2.4. Influence of Matrine on Biological Characteristics of Akanthomyces attenuatus

Five concentrations of matrine (1.0, 0.5, 0.25, 0.125, and 0.05 mg/L) were individually added to sterilized SDA broth (50 mL) followed by inoculation of *A. attenuatus* conidial suspension (1 × 10^4^ conidia/mL; 2 mL). Culture medium containing sterile water without matrine served as control. Cultures were incubated at 150 rpm and 23 ± 2 °C for 5 days. Fungal culture samples (1 mL) were taken from each treatment every 24 h and observed under a compound microscope (Ningbo Shunning Instruments Co. Ltd., Guangzhou, China) to count the total number of germinated conidia following the method of Wu et al. [12]. The whole experimental setup was performed three times using freshly prepared fungal suspension.

Matrine (1.0, 0.5, 0.25, 0.125, and 0.05 mg/L) was individually layered upon solidified PDA medium followed by overnight drying. PDA plates without matrine served as controls. PDA plates were then inoculated with mycelial plugs (2 cm diameter) of *A. attenuatus* followed by incubation at 26 °C, 80 ± 5% relative humidity and 16 L: 8 D photoperiod for 7 days. Colony growth data was obtained, as outlined by Wu et al. [12], at 24 h intervals.

### 2.5. Bioassay Studies

#### 2.5.1. Bioassay 1: Efficacy of *Akanthomyces attenuatus* Isolate SCAUDCL-53 against *Megalurothrips usitatus*

The efficacy of *A. attenuatus* against *M. usitatus* was studied by following the centrifuge tube residual method of Du et al. [7] and Yang et al. [3]. Briefly, bean pods (1 cm length) and centrifuge tubes (9 cm length) were individually immersed in different conidial concentrations (1 × 10^4^, 1 × 10^5^, 1 × 10^6^, 1 × 10^7^ and 1 × 10^8^ conidial/mL) for 2 h followed by air drying. Bean pods and centrifuge tubes immersed in ddH_2_O served as control. One hundred individuals of *M. usitatus* adult females were then transferred to centrifuge tubes, and bean pods were treated with the same concentration. The tubes were sealed with a cotton plug. The centrifuge tube was then incubated at 26 °C, 80 ± 5% relative humidity and 16 L: 8 D photoperiod. The whole study was performed three times using freshly prepared fungal concentrations and fresh batches of insects. Mortality data was collected every 24 h as outlined by Du et al. [7].

#### 2.5.2. Bioassay 2: Efficacy of Matrine against *Megalurothrips usitatus*

The efficacy of five matrine concentrations (1.0, 0.5, 0.25, 0.125, and 0.05 mg/L) against *M. usitatus* under laboratory conditions was investigated. Briefly, bean pods (1 cm length) and centrifuge tubes (9 cm length) were individually immersed in different matrine concentrations for 2 h followed by drying under sterilized conditions. Bean pods and centrifuge tubes immersed in ddH_2_O served as control. One hundred individuals of *M. usitatus* adult females were transferred to the centrifuge tubes, and bean pods were treated with the same concentration. The tubes were then sealed with a cotton plug. The centrifuge tube was then incubated at 26 °C, 80 ± 5% relative humidity and 16 L: 8 D photoperiod. The whole study was performed three times using freshly prepared fungal concentrations and fresh batches of insects. Mortality data was collected every 24 h for 5 days.

#### 2.5.3. Bioassay 3: Efficacy of Single or Joint Treatments of *Akanthomyces attenuatus* and Matrine against *Megalurothrips usitatus*

Efficacy of single or joint treatments of *A. attenuatus* and matrine alone or in combination with each other was studied against *M. usitatus* adult females following the centrifuge tube residual method described in bioassays 1 and 2.

### 2.6. Enzymatic Activity of Megalurothrips usitatus in Response to Single or Joint Treatments of Akanthomyces attenuatus and Matrine

#### 2.6.1. Insect Treatment and Sample Preparation

*Megalurothrips usitatus* adult females (60 females) treated with *A. attenuatus* (1 × 10^5^ conidia/mL), matrine (0.5 mg/mL) and their combination (*A. attenuatus* 1 × 10^5^ conidia/mL + matrine 0.5 mg/mL), following the centrifuge tube residual method described in bioassays 1 and 2, were collected after 3 and 5 days of treatment. Collected insects were homogenized in ice cold 0.05 M potassium phosphate buffer (PBS) for the enzyme assays following the method of Ali et al. [22].

#### 2.6.2. Total Protein Assay

Total protein concentration of insect samples was determined by the Bradford assay [25].

#### 2.6.3. Enzyme Activity Assays

The activity of Acetylcholinesterase (AChE) of *M. usitatus* samples was determined following the method of Ellman et al. [26]. The changes in absorbance of reaction mixture (consisting of 50 μL sample solution, 100 μL 45 µM 5-5-dithiobis-(2-nitrobenzoic acid), 100 µL acetylthiocholine iodide and 90 µL sodium phosphate buffer) were recorded at 405 nm for 40 min. One unit of enzyme activity was described as change in absorbance per min per mg protein (mOD/min/mg).

Superoxide dismutase (SOD) assay was carried out following the method of Beauchamp and Fridovich [27]. One unit of enzyme activity was described as the amount of SOD required for 50% inhibition of nitro blue tetrazolium reduction reaction per mg protein at 560 nm.

Catalase activity was assessed via spectrophotometric analysis of hydrogen peroxide (H_2_O_2_) decomposition at 240 nm [28]. One unit of catalase activity was defined as the amount of enzyme that decomposes 1 mmol H_2_O_2_/min at an initial H_2_O_2_ concentration of 30 mM at pH 7.0 and 25 °C.

Peroxidase (POD) assays were carried out following the method of Shannon et al. [29]. Briefly, 0.1 mL of 14 mM potassium phosphate buffer and 0.1 mL of sample were added to the reaction mixture incubated at 20 °C for 10 min followed by mixing through inversion. Changes in absorbance were measured at the wavelength of 420 nm.

### 2.7. Data Analysis

Fungal germination and growth data were analyzed through one-way ANOVA, and significant means were compared by Tukey’s HSD test (*p* <0.05).

The synergistic interaction between *A. attenuatus* and matrine was calculated by following the equation below from Wu et al. [12].

$$Corrected\;mortality\;\left(M\right)=\frac{\left(Mortality\;in\;response\;to\;treatment-Mortality\;in\;response\;to\;control\right)}{\left(1-Mortality\;in\;response\;to\;control\right)}$$

$$Expected\;mortality\;\left(M_{e}\right)=M_{A}+M_{B}*\left(1-M_{A}\right)$$

$$Chi\;square\;\left(\chi^{2}\right)=\frac{\left(M_{AB}-M_{e}\right)*100*\left(M_{AB}-M_{e}\right)}{\left(M_{e}\right)}$$

## 3. Results

### 3.1. Influence of Matrine on Biological Characteristics of Akanthomyces attenuatus

**Figure 1:**
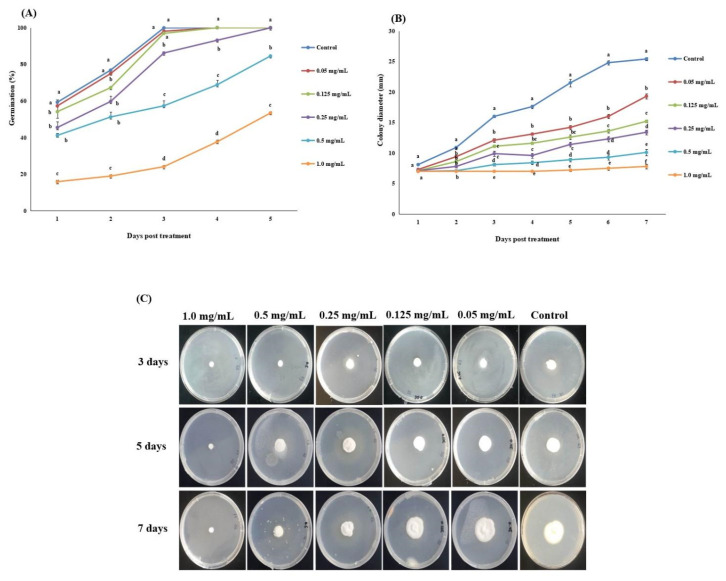
Influence of matrine biological characteristics of *A. attenuatus.* (**A**) Germination rate (%); (**B**) Colony diameter; (**C**) Growth of *A. attenuatus* on PDA plates supplemented with different concentrations of matrine. Error bars indicate standard error of means based on three replicates. Bars having different letters at different days post treatment were significantly different from each other.

The germination rate (%) of *A. attenuatus* was significantly affected by different matrine concentrations when compared to the control (Figure 1A). After 5 days of growth, the lowest rate of germination (15.9%) was observed for 1.00 mg/L matrine concentration, whereas the highest germination rate was recorded from the control (Figure 1A).

Different matrine concentrations significantly affected the radial growth of *A. attenuatus* when compared with the control (Figure 1B,C). The average colony diameter decreased with an increase in concentration of matrine. After seven days of growth, the highest radial growth (25.4 mm) was recorded from the control, whereas the lowest colony diameter was observed for 1.00 mg/L matrine which had a mean value of 7.8 mm (Figure 1B).

### 3.2. Bioassay Studies

#### 3.2.1. Bioassay 1: Efficacy of *Akanthomyces attenuatus* against *Megalurothrips usitatus*

**Figure 2:**
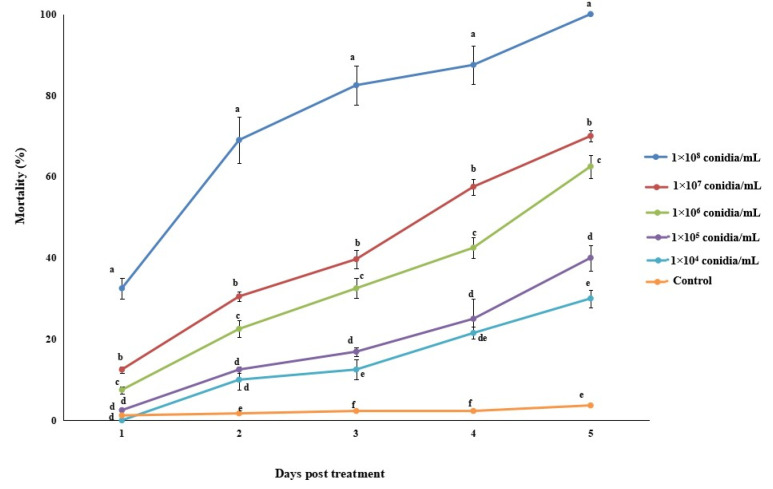
Percentage mortality of *M. usitatus* in response to *A. attenuatus* applications at different time intervals. Error bars indicate standard error of means based on three replicates. Bars having different letters at different days post treatment were significantly different from each other.

*Megalurothrips usitatus* adult females treated with five different conidial concentrations of *A. attenuatus* had different rates of mortality at different time intervals (Figure 2). The highest mortality at different time intervals post fungal treatment was observed for 1 × 10^8^ conidia/mL. After five days of treatment, the LC_50_ of *A. attenuatus* was 2.14 × 10^5^ conidia/mL.

#### 3.2.2. Bioassay 2: Efficacy of Matrine against *Megalurothrips usitatus*

**Figure 3:**
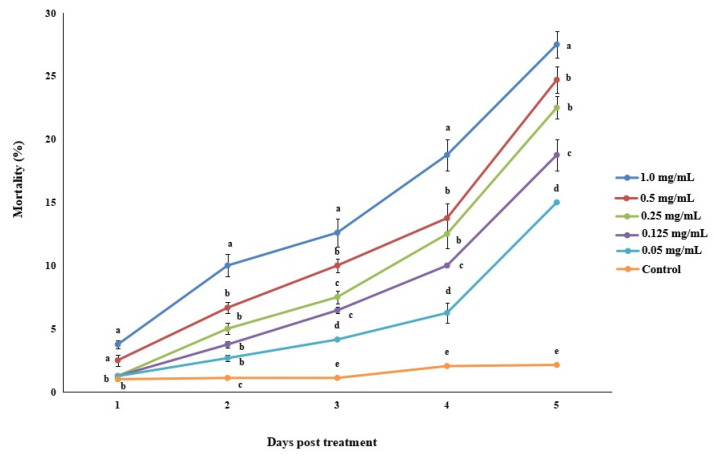
Percentage mortality of *M. usitatus* treated with different concentrations of matrine at different time intervals. Error bars indicate standard error of means based on three replicates. Bars having different letters at different days post treatment were significantly different from each other.

Significantly different rates of *M. usitatus* mortality were observed for different matrine concentrations when compared with the control. After 5 days of treatment, the mortalities of *M. usitatus* treated with 0.5 and 1.0 mg/mL matrine were 24.7 and 27.5%, respectively (Figure 3). After five days of treatment, the LC_50_ of matrine was 5.4 mg/mL.

#### 3.2.3. Bioassay 3: Efficacy of Single or Joint Treatments of *Akanthomyces attenuatus* and Matrine against *Megalurothrips usitatus*

The combined treatments of *A. attenuatus* and matrine caused significantly higher *M. usitatus* mortality compared to the control and their respective individual treatments (Table 1). After five days of treatment, all concentrations of matrine combined with different concentrations of *A. attenuatus,* except one combination (matrine 0.25 mg/mL + 1 × 10^7^ conidia/mL), showed a synergistic effect. The mortality caused by different combinations were higher than 60%, among which the combination of matrine at 0.5 mg/mL and *A. attenuatus* at 1 × 10^7^ conidia/mL had the highest morality (89.67%).

### 3.3. Enzymatic Response of Megalurothrips usitatus to Individual or Combined Treatments of Akanthomyces attenuatus and Matrine

**Figure 4:**
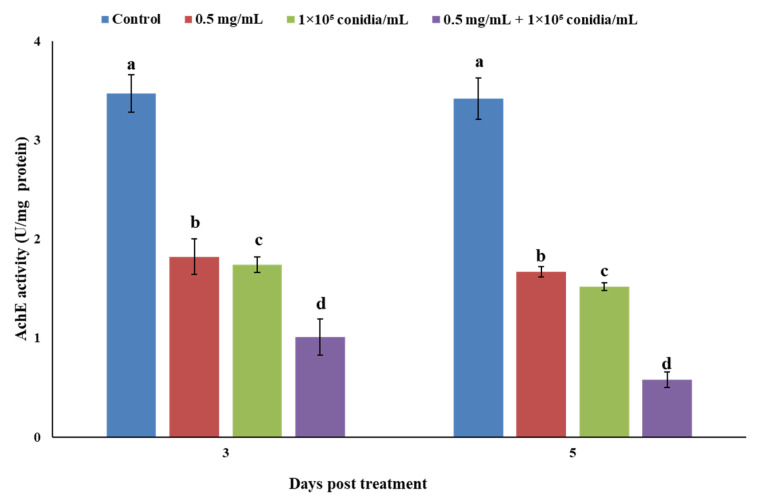
Acetylcholinesterase (AChE) activity of *M. usitatus* in response to individual or joint treatments of *A. attenuatus* and matrine. Bars having different letters show significant differences between treatments at different time intervals.

Acetylcholinesterase activity of *M. usitatus* was significantly reduced among different treatments and after 3 and 5 days of treatment. The results indicated that AChE activity observed at the end of the experimental period were significantly lower than 3 days post treatment (Figure 4).

**Figure 5:**
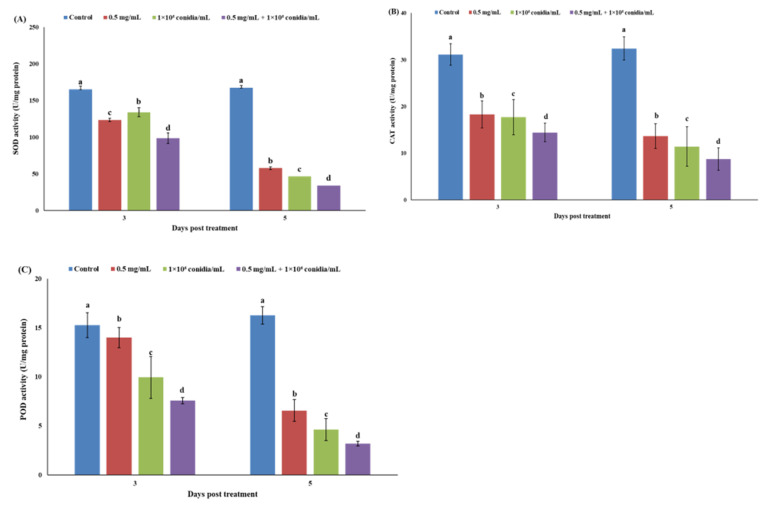
Changes in antioxidant enzyme activities of *M. usitatus* following single or joint treatments of *A. attenuatus* and matrine after 3 and 5 days of treatment. (**A**) Superoxide dismutase (SOD) activity; (**B**) Catalase (CAT) activity; (**C**) Peroxidase (POD) activity. Bars having different letters show significant differences between treatments at different time intervals.

Antioxidant enzyme (superoxide dismutase, catalase and peroxidase) activities of *M. usitatus* were significantly reduced among different treatments after 3 and 5 days of treatment. Activities of antioxidant enzymes superoxide dismutase, catalase and peroxidase in response to different treatments after 3 days were higher than 5 days post treatment (Figure 5A–C).

## 4. Discussion

A detailed knowledge concerning the influence of chemical insecticides on growth virulence and enzymatic activities is a pre-requisite in developing integrated control measures based on insect pathogenic fungi and chemical pesticides [12]. Previous studies on *A. attenuatus* have only studied its pathogenicity against *M. usitatus* [3,7]. Very few laboratory studies are available to date which can explain the possible synergistic, antagonistic or additive effects of joint formulations of *A. attenuatus* and chemical pesticides against *M. usitatus.* This study explains a possible synergistic interaction between *A. attenuatus* and matrine against *M. usitatus* as well as effects of this synergism on activities of detoxifying enzymes within *M. usitatus.*

Our findings on the influence of different matrine concentrations on the biological characteristics of *A. attenuatus* revealed a significant reduction in germination and radial growth when compared to controls. These results are consistent with Wu et al. [12] who observed similar effects of matrine influencing biological characteristics on *Beauveria brongniartii* Sacc. (Hypocreales; Cordycipitaceae). Our results are also consistent with Xu et al. [30] who showed that germination and radial growth of *Isaria fumosorosea* Wize (Hypocreales; Cordycipitaceae) was inhibited by 20-Hydroxyecdysone.

Our results revealed the susceptibility of *M. usitatus* adult females to *A. attenuatus.* The LC_50_ of *A. attenuatus* observed in this study (2.1 × 10^5^ conidia/mL) was lower from the LC_50_ *A. attenuatus* isolates SCAUDCL-38 (1.9 × 10^6^ conidia/mL) observed by Du et al. [7]. The toxicity of different matrine concentrations was also tested against *M. usitatus* adult females. Based on mortality data, LC_50_ of matrine against *M. usitatus* was 5.4 mg/mL which is different to the LC_50_ of matrine against *Bemisia tabaci* Gennadius (Hemiptera; Aleyrodidae) as reported by Ali et al. [22].

Matrine has shown synergistic interaction with insect pathogenic fungi against different insect pests [12,22,31]. Our results revealed a strong synergistic effect between *A. attenuatus* and matrine against *M. usitatus.* The levels of synergism observed here are similar with that recorded by Ali et al. [22] who observed enhanced mortalities of whitefly treated with different combinations of matrine and *A. muscarium.* The possible synergistic action between both control agents was due to them both working on the same target site; matrine and *A. attenuatus* both attack insect acetylcholine receptors [32,33,34,35].

These findings also revealed the possible physiological and biochemical effects of combined *A. attenuatus* and matrine treatments on *M. usitatus.* The results of enzyme quantification assays revealed significant fluctuation following *A. attenuatus* and matrine treatments alone or in combination with each other. Acetylcholinesterase (AChE) is an important enzyme of an insect’s central nervous system, being involved in the termination of nerve impulses at peripheral or central synapses through hydrolysis of acetylcholine [23,36]. In this study, AchE activities of *M. usitatus* in response to different treatments were significantly lower than the control. The decrease in AchE activity is related to the common site of action within the insect host. Matrine, an alkaloid extracted from *S. flavescens*, is known to target insect acetylcholine receptors which in turn affects AchE production [36]. Ali et al. [22] observed a similar reduction in AchE activities of *B. tabaci* in response to matrine application. The reduction in AchE activities of *M. usitatus* can be related to the production of the secondary metabolite named bassianolide by fungi belonging to the genus *Akanthomyces* and *Beauveria* which is known to inhibit AchE activities in insects [17,25,26].

Antioxidant enzymes (superoxide dismutase, catalase and peroxidase) are cellular system enzymes which eliminate reactive oxygen species (ROS) involved in the reduction of bio-membrane damage [37]. Superoxide dismutase, catalase and peroxidase activities, in response to different treatments after 3 days, were higher than 5 days post treatment. The inhibition of these enzymes across the experimental period meant reduced elimination of ROS and denaturation of different biomolecules from the insect body leading to insect death [31,38].

## 5. Conclusions

*Megalurothrips usitatus* adult females were susceptible to individual or combined treatments of *A. attenuatus* and matrine. Our results report a strong synergistic interaction between *A. attenuatus* and matrine against *M. usitatus* under laboratory conditions. The enzyme response of *M. usitatus* to individual or combined treatments of *A. attenuatus* and matrine further explained the biochemical nature of the synergistic effect. Our results will serve as basic information for designing *A. attenuatus* and matrine-based *M. usitatus* management programs in the future, although further studies on the sublethal effects of these control agents against *M. usitatus* are required.

## Figures and Tables

**Table 1:** Mean percentage adjusted mortality of *M. usitatus* following single or joint treatments of *A. attenuatus* and matrine.

Treatments		*Megalurothrips usitatus* Mortality (%) at Different Time Intervals				
		1d	2d	3d	4d	5d
Matrine (mg/mL)	0.50	2.5 ± 0.36 d	6.67 ± 0.67 g	10.23 ± 0.45 g	13.75 ± 1.24 i	24.70 ± 1.89 h
	0.25	1.25 ± 0.14 e	5.12 ± 0.23 g	7.53 ± 0.35 h	12.51 ± 1.21 jk	22.51 ± 1.23 i
*Akanthomyces attenuatus* (conidia/mL)	1 × 10^7^	12.54 ± 0.89 b	30.36 ± 1.49 b	39.67 ± 4.41 d	57.48 ± 1.61 h	70.03 ± 1.34 d
	1 × 10^6^	7.51 ± 1.67 d	22.48 ± 1.21 c	32.51 ± 3.21 d	42.47± 2.09 ij	62.48 ± 1.37 ef
	1 × 10^5^	2.47 ± 0.17 d	9.98 ± 0.23 fg	12.56 ± 1.67 f	25.07± 1.67 j	40.12 ± 2.89 g
Matrine (mg/mL) + *Akanthomyces attenuatus* (conidia/mL)	0.50 + 1 × 10^7^	17.23 * ± 2.33 a (9.59;17.19)	36.67 * ± 1.67 a (25.49;21.00)	51.34 * ± 3.14 a (35.56;3.80)	72.33 * ± 3.12 b (53.63;5.54)	89.67 * ± 2.88 a (75.09; 4.83)
	0.25 + 1 × 10^7^	12.67 ± 1.67 b (8.16;0.02)	30 * ± 2.89 b (16.00;12.24)	46.03 * ± 2.12 b (26.39;14.56)	67.67 * ± 6.11 bc (42.66;14.65)	85.00 ± 2.89 ab (71.25;1.43)
	0.50 + 1 × 10^6^	8.33 ± 0.33 c (6.55;0.33)	21.67 ± 3.33 c (14.44;1.27)	39.67 * ± 2.12 c (36.39;2.41)	69.67 *± 3.13 bc (50.37;7.38)	86.67 *± 5.14 ab (56.01;16.77)
	0.25 + 1 × 10^6^	8.33 ± 1.67 c (6.55;0.48)	11.67 ± 1.12 e (12.89;0.11)	26.67 ± 1.67 e (21.98;1.01)	58.33 *± 2.89 d (49.68;11.50)	81.33 *± 3.12 b (70.93;25.96)
	0.50 + 1 × 10^5^	5 ± 1.33 cd (4.93;0.00)	16.67 ± 3.33 d (15.94;0.03)	32.33 *± 1.12 d (21.25;5.77)	66.67 *± 2.89 c (35.27;27.94)	78.33 *± 2.12 c (54.82;10.08)
	0.25 + 1 × 10^5^	3.33 ± 1.67 d (3.71;0.04)	11.67 * ± 2.89 e (6.55;4.01)	25.00 * ± 5.00 c (15.97;5.11)	56.67 *± 4.33 d (34.37;14.46)	66.67 *± 3.14 e (53.5;8.24)

Means (±SE) followed by different letters are significantly different (Tukeys’s *p* < 0.05). Data in bracket shows the expected mortality and χ^2^-value, respectively. * represents the synergistic interaction.

## Data Availability

The raw data supporting the conclusion will be made available by the corresponding author on request.
